# Bicuspid valve aortopathy is associated with distinct patterns of matrix degradation

**DOI:** 10.1016/j.jtcvs.2019.08.094

**Published:** 2020-12

**Authors:** Ya Hua Chim, Hannah A. Davies, David Mason, Omar Nawaytou, Mark Field, Jillian Madine, Riaz Akhtar

**Affiliations:** a Department of Mechanical, Materials and Aerospace Engineering, School of Engineering, University of Liverpool, Liverpool, United Kingdom; b Institute of Integrative Biology, Faculty of Health and Life Sciences, University of Liverpool, Liverpool, United Kingdom; c Liverpool Centre for Cardiovascular Sciences, University of Liverpool, Liverpool, United Kingdom; d Department of Cardiac Surgery, Liverpool Heart and Chest Hospital, Liverpool, United Kingdom

**Keywords:** bicuspid aortic valve aortopathy, elastin, microstructure, idiopathic degenerative aneurysms, micromechanics, biochemistry, AI, aortic insufficiency, AS, aortic stenosis, AsAA, ascending aortic aneurysm, BAV, bicuspid aortic valve, BAV-A, bicuspid aortic valve aneurysm, CABG, coronary artery bypass graft, DA, idiopathic aortic aneurysm, *E*, elastic modulus, GAG, glycosaminoglycan, M_1_, inner media, M_2_, middle media, M_3_, outer media, MMP, matrix metalloproteinase, TAV, tricuspid aortic valve

## Abstract

**Objective:**

To explore the micromechanical, biochemical, and microstructural differences between bicuspid aortic valve aneurysm (BAV-A) and tricuspid aortic valve idiopathic degenerative aneurysm (DA), compared with normal aorta.

**Methods:**

Aortic tissue was obtained from patients undergoing aneurysmal repair surgery (BAV-A; n = 15 and DA; n = 15). Control tissue was obtained from aortic punch biopsies during coronary artery bypass graft surgery (n = 9). Nanoindentation was used to determine the elastic modulus on the medial layer. Glycosaminoglycan, collagen, and elastin levels were measured using biochemical assays. Verhoeff Van Gieson–stained cross-sections were imaged for elastin microstructural quantification.

**Results:**

The elastic modulus was more than 20% greater for BAV-A relative to control and DA (signifying a loss of compliance). No significance difference between control and DA were observed. Collagen levels for BAV-A (36.9 ± 7.4 μg/mg) and DA (49.9 ± 10.9 μg/mg) were greater compared with the control (30.2 ± 13.1 μg/mg). Glycosaminoglycan and elastin levels were not significant between the groups. Elastin segments were uniform throughout the control. Aneurysmal tissues had less elastin segments close to the intima and adventitia layers. Both BAV-A and DA had elastin segments compacted in the media; however, elastin segments were highly fragmented in DA.

**Conclusions:**

BAV-A has a greater loss of aortic wall compliance relative to DA and the control. Although elastin levels were equal for all groups, spatial distribution of elastin provided a unique profile of matrix degradation for BAV-A. Elastin compaction within the media of BAV-A may have resulted from the altered hemodynamic pressure against the wall, which could explain for the stiffness of the tissue.

## Graphical abstract

**Figure:**
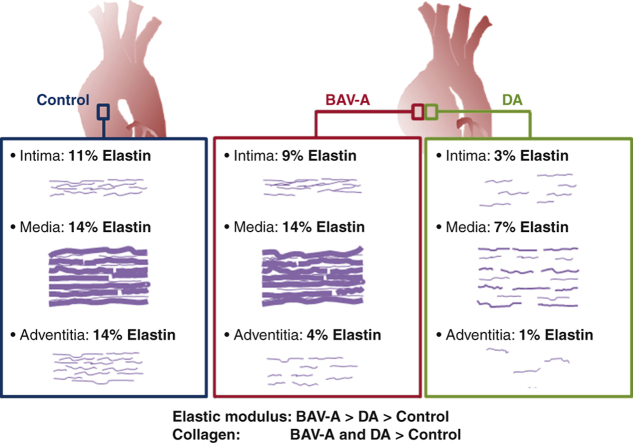

**Figure:**
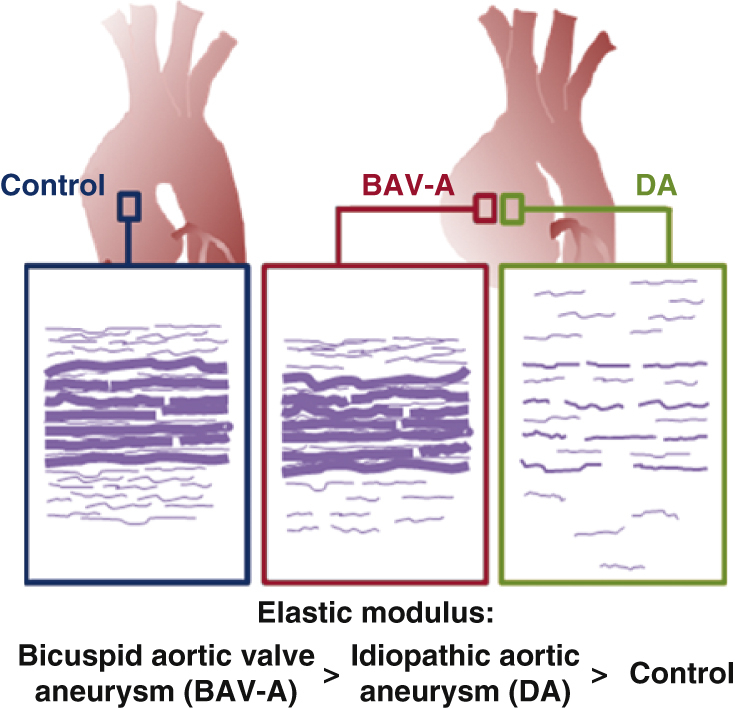
BAV-A is stiffer relative to DA, at a >5-cm aorta diameter.

**Central Message**

Bicuspid aortic valve aneurysm (BAV-A) patients display a stiffer aortic wall relative to idiopathic aortic aneurysm (DA) patients. This is related to elastin microstructure rather than elastin level.

**Perspective**

This study focuses on the microstructural properties of the aorta in BAV-A and DA compared with controls. Elastin levels were comparable; however, spatial distribution of elastin fibers differed between each group. BAV-A was stiffer compared with DA and controls. Using our approach on different subsets of BAV-A may reveal whether lower-diameter threshold patients are at an increased risk of rupture.

See Commentaries on pages e259 and e261.

Bicuspid aortic valve (BAV) is associated with aortic dilation,^1^ with up to 50% of patients with BAV developing ascending aortic aneurysms (AsAAs).^2^ This is attributed to altered hemodynamics resulting in an eccentric helical flow pattern.^3^^,^^4^ Hemodynamics coupled with genetic or developmental defects are implicated in aberrant remodeling of the aortic wall relative to individuals with a tricuspid aortic valve (TAV).^4^ This appears to manifest in microstructural organization that is distinct to patients with BAV. For instance, elastin and collagen fibers become more highly aligned in patients with BAV and AsAA than in patients with TAV and AsAA.^5^ Despite more understanding of bicuspid aortic valve aneurysms (BAV-As), significant gaps in knowledge remain, hampering optimal clinical management for these patients.^1^

Conventionally, patients with BAV were recommended for aggressive surgical treatment due to concerns that the aortic wall may be vulnerable to dissection, mirroring the approach taken for connective tissue disorders such as Marfan syndrome.^6^ Recently, a more conservative clinical stance has been proposed.^6^^,^^7^

In the BAV aortic wall, medial degeneration including elastic fiber fragmentation has been reported even in nondilated aortas,^1^^,^^8^^,^^9^ similar to idiopathic degenerative aneurysms (DAs).^10^ Biomechanical tests on BAV and DA tissue from patients with a similar preoperative aortic diameter exhibited relatively similar properties.^11^ However, there are significant gaps in the literature, as detailed mechanical and biochemical characterization comparing BAV-A and DA directly has not been performed.

Here, we hypothesized that unique patterns of aortic wall degradation could be identified in BAV-A relative to DA, for an equivalent aortic diameter, and that both would be distinct from normal aortic tissue. We investigated the association between micromechanical properties, structural proteins (collagen, and elastin), and glycosaminoglycan (GAG) levels between patients with BAV-A and DA in the ascending aorta. Control aortic tissue was taken from patients undergoing coronary artery bypass graft (CABG) operations. Subsequently, we characterized elastin microarchitecture to identify distinct matrix profiles amongst the groups.

## Methods

### Human Aortic Tissue

Ascending aortic tissue (mainly from the greater curvature) was collected from 39 patients who provided consent and who were undergoing CABG for vein graft proximal anastomosis (n = 9) and elective aneurysmal repair of AsAA according to American Heart Association guidelines^12^ (BAV-A; n = 15 and DA; n = 15) at Liverpool Heart and Chest Hospital. Table 1 shows patient clinical data, pathology information, and procedure performed for each group. For the CABG group, n = 8 for each test (of the 9 available) (Table E1). Patients with DA were defined as idiopathic if they had a TAV with no clinical characteristics, family history, or if genetic testing did not detect connective tissue disorders. Patients with a known connective tissue disorder were excluded. Patients with BAV-A were identified intraoperatively or via presurgical echocardiogram. Surgery was performed once the preoperative aortic diameter reached 5.5 cm unless there were other indicators. In total, 11 of 15 patients with BAV-A underwent surgery at diameters <5.5 cm due to symptomatic aortic valve disease with severe stenosis/regurgitation or both. In total, 6 of 15 patients with DA underwent surgery at diameters <5.5 cm because 1 had acute presentation with pain, 1 had rapid increase in size >5 mm in 1 year, and 4 had symptomatic aortic valve disease with severe stenosis/regurgitation or both.

**Table 1:** Aortic pathology, patient clinical characteristics, and procedures performed for the patients used in this study, grouped by clinical condition

Patient characteristics	Control	BAV-A	DA	*P* value
Pathology: etiology				
Normal: coronary artery disease, n	9			
Aneurysm; bicuspid syndrome, n		15		
Aneurysm: idiopathic degenerative, n			15	
Clinical characteristics				
Age, y	60.0 ± 8.9	61.0 ± 11.3	66.4 ± 13.4	.331
Sex				
Male, n	7	7	8	.315
Female, n	2	8	7	
BMI, kg/m^2^	28.5 ± 5.8	28.8 ± 7.5	27.5 ± 5.5	.844
Preoperative aortic diameter, cm	3.4 ± 0.2	5.1 ± 0.6	5.2 ± 1.4	.000262
Indication for surgery, <5.5 cm	3.4 ± 0.2	4.8 ± 0.4	4.4 ± 0.7	.00000463
n	9	11	9	
Hypertension				
Yes, n	5	8	11	.486
No, n	4	7	4	
Cholesterol				
Yes, n	6	6	10	.263
No, n	3	9	5	
Family history aneurysm				
Yes, n	1	2	2	.985
No, n	8	13	13	
Procedure performed				
CABG, n	9			
AVR, ARR, replacement of ascending aorta and part of arch, n		3	2	
AVR and ARR, n		0	1	
ARR and replacement of ascending aorta, n		4	4	
Replacement of ascending aorta, n		5	4	
Replacement of ascending aorta and part of arch, n		2	2	
TAR and replacement of ascending aorta, n		0	2	
TAR, n		1	0	

Data are displayed as mean ± standard deviation; n represents the number of patients. *P* value for analysis of variance and χ^2^ test. *BAV-A*, Bicuspid aortic valve aneurysm; *DA*, degenerative aneurysm; *BMI*, body mass index; *CABG*, coronary artery bypass graft, *AVR*, aortic valve replacement, *ARR*, aortic root replacement, *TAR*, total arch replacement.

Of the patients with BAV-A, 1 patient had a normal valve function, 2 had aortic insufficiency (AI), 7 had aortic stenosis (AS), and 5 had a mixture of AI and AS. Our patients were not indexed to body size. The resected tissue was cut into segments and processed as follows: for micromechanical and biochemical experiments, samples were rapidly frozen in dry ice and isopentane slurry, and immediately frozen at –80°C until testing. For histology, the tissue was formalin-fixed and paraffin-embedded. Full ethical approval was obtained through the LBIH Biobank (project numbers 15-06 and 18-07). The LBIH Biobank confers ethical approval for the use of samples through their ethical approval as a Research Tissue Bank (REC reference 14/NW/1212, NRES Committee North West–Haydock).

### Nanoindentation

**Figure 1:**
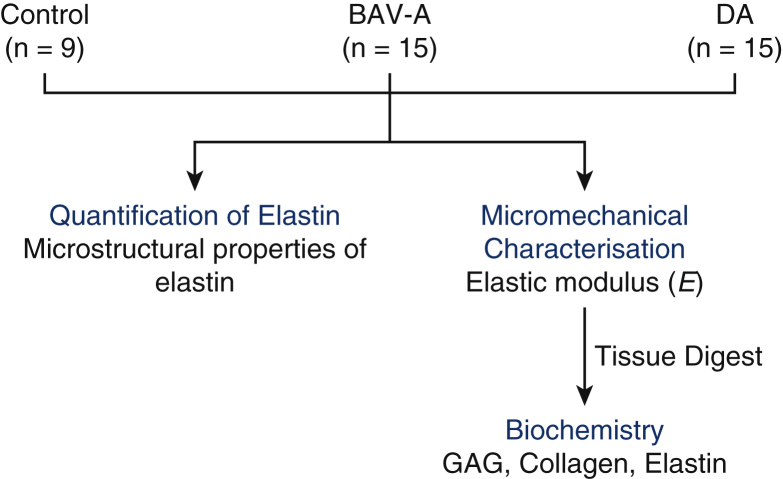
Workflow for the study. Aortic tissue from control, BAV-A, and tricuspid aortic valve idiopathic DA patients were used for micromechanical characterization. Using oscillatory nanoindentation, we measured the elastic modulus (*E*) at the medial layer of the tissue. Subsequently, all tissues were fully digested for biochemistry. Levels of GAG, collagen, and elastin were quantified. Using the same tissue cohorts, Verhoeff Van Gieson–stained cross-sections were used to characterize elastin microstructure. From the acquired images, we measured elastin lamellar units, lamellar spacing, and elastic lamellae thickness, over the media. Furthermore, tissue thickness, elastin content, number of elastin segments, and the length of the segments were also measured over the entire tissue cross-section. *BAV-A*, Bicuspid aortic valve aneurysm; *DA*, degenerative aneurysm; *GAG*, glycosaminoglycan.

Oscillatory nanoindentation was used to determine the elastic modulus (*E*) of the tissues.^13^ *E* describes the ability of an elastic material to resist deformation to an applied stress, providing an indication of localized tissue compliance. Nanoindentation was performed on 3 specimens/patient for aneurysmal tissues and 1 specimen/patient for controls. Samples were removed from –80°C, thawed at room temperature, and cut into ∼0.5-cm wide strips for the aneurysmal tissues. Control punch biopsies (0.5 cm in diameter) were cut at opposite edges to expose flat surfaces. A Nanoindenter G200 with a DCM-II actuator (KLA-Tencor, Milpitas, Calif) with a 100-μm flat punch indenter (Synton-MDP Ltd, Nidau, Switzerland) was used. Sixteen indents were applied to the medial layer, indenting the tissue cross-section. All measurements were performed using a precompression of 7 μm, at a frequency of 110 Hz, with 500-nm oscillation amplitude. Full methodological details have been presented elsewhere.^13^ The specimens were kept hydrated in phosphate-buffered saline and all tests performed at 22°C. Post-measurements, the specimens were refrozen for biochemical analysis on the same samples (Figure 1).

### Biochemical Assays

GAG, collagen, and elastin levels were quantified using biochemical assays. Details of methods, reagents used, and their concentrations are presented in Appendix E1. Aortic specimens were digested by either papain solution to extract for GAGs and collagen, or oxalic acid to extract for elastin. All papain-digested specimens were assayed as triplicates, whereas oxalic acid–digested specimens were assayed as duplicates. Dimethyl methylene blue dye was used to detect sulfate GAG levels within the tissue and subsequently compared against the standard; chondroitin sulfate C.^14^ Collagen levels were determined by the concentration of hydroxyproline within the tissue, and compared with L-hydroxyproline.^15^ Finally, following the Fastin Elastin Kit (Biocolor, Carrickfergus, UK) protocol, α-elastin levels within the tissue were detected.

### Histology

Paraffin-embedded samples were sectioned to 6 μm thickness and Verhoeff-Van Gieson stain was used (Elastic Stain Kit; Sigma-Aldrich, St Louis, Mo). The stained sections were examined on a Nikon Eclipse Ci microscope (DS-Fi2 camera), and observed using a 40× 0.75 NA objective (Nikon, Tokyo, Japan). Images were taken across the tissue cross-section and stitched with NIS Elements software (v4.13.03; Nikon). Four random cross-sectional stitched images of the entire wall per tissue were taken, with 2 tissue sections per patient imaged where possible.

### Image Processing

**Figure 2:**
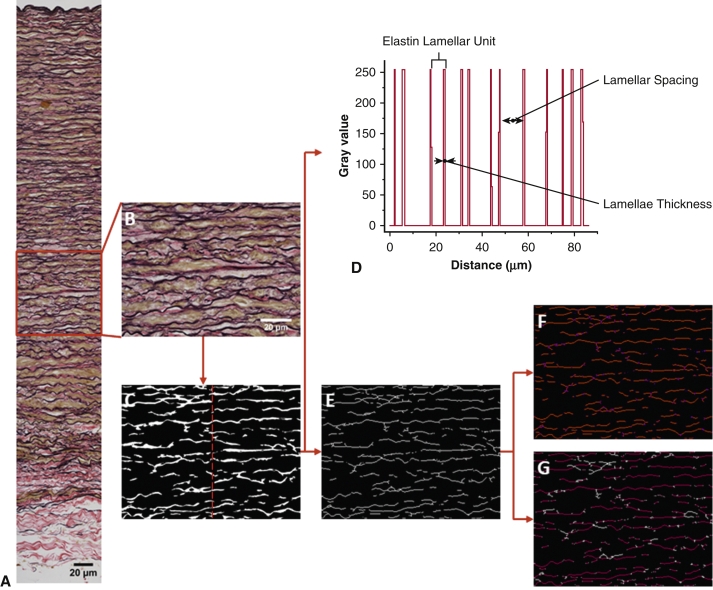
Image processing procedure to obtain microstructural properties of elastin. A, Representative stitched Verhoeff Van Gieson–stained aortic wall image. B, Middle media segment of the tissue highlighted was used in pixel classification. Pixel classifier was trained using 6 images before (C) simple segmented mask image was exported. Typically to quantify elastic lamellae properties in the media, a line was drawn at the area of interest like the *red central line* drawn in (C) to produce (D) a plot profile, and subsequently the number of lamellar units, lamellar spacing, and elastic lamellae thickness were measured. To quantify elastin segments over the entire tissue cross-section, in E, a downscaled and skeletonized image of the mask image was required. Our in-house macro script identifies F, the number of elastin segments (*red lines* separated out with *purple ends*) and G, the length of elastin segments (between the *white ends*). All images were taken at 40× magnification. Scale bar: 20 μm.

Figure 2 shows the procedure for quantifying the stitched images. The elastic fibers were segmented using Ilastik software (v1.3.0; https://www.ilastik.org/). All segment masked images were subsequently analyzed using Fiji software (v1.52e; https://imagej.net/Fiji/Downloads). Before the quantifications, the images were portioned into 10 sections to cover innermost media, inner media (M_1_), middle media (M_2_), outer media (M_3_), and adventitia (Figure 2, *A*). The following parameters were measured: layer thickness, elastin content of the portioned sections, number of medial lamellar units, lamellae spacing within the units, the thickness of the lamellae, number of elastin segments from each portioned section, and the length of these segments. Further details are in Appendix E1. The measured segments include both elastic lamellae and interlamellar fibers. Hence, the elastin segment length represents complete lengths of elastin (lamellar fibers and finer interlamellar elastin fibers) identified per image.

### Statistical Analysis

Data were analyzed using OriginPro 2016 (OriginLab Corporation, Northampton, Mass). Median values for each patient are presented using box plots with 5th and 95th percentile as whiskers, and data overlaid. All data were non-normally distributed as shown in Q-Q plots made from the data before statistical analysis (not shown in the paper). For all comparisons between all 3 groups, the Kruskal–Wallis test and post hoc analysis according to the Dunn test for multiple comparisons was performed at a significance level of .05.

## Results

### Comparable Clinical Demographics in BAV-A and DA

Table 1 presents clinical characteristics for all patients. For the quantitative data, both aneurysmal cohorts were matched by age (*P* = .07), preoperative aortic diameter (*P* = .85), and body mass index (*P* = .84). Controls were matched with both aneurysmal groups by body mass index (*P* = .87 for BAV-A and DA), and only with BAV-A by age (*P* = .49). Preoperative aortic diameter was significantly different between the control and both aneurysmal groups (*P* < .001 for BAV-A and DA). The percentage split among the groups for the categorical data were as follows: male, 78% (control), 47% (BAV-A), and 53% (DA); hypertensive, 56% (control), 53% (BAV-A), and 73% (DA); cholesterol-medicated patients, 67% (control), 40% (BAV-A), and 67% (DA); and family history of aneurysm, 11% (control) and 15% (BAV-A and DA).

### Homogenous Micromechanical–Biochemical Properties in BAV-A

**Figure 3:**
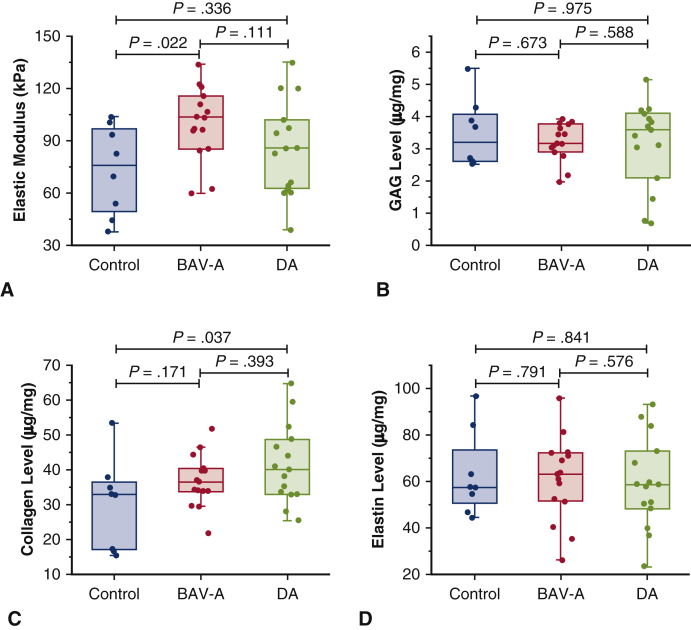
Micromechanical and biochemical data for control (n = 8), BAV-A (n = 15), and tricuspid aortic valve idiopathic DA (n = 15). All data was represented as box plots and data overlaid with *upper* and *lower borders* of the box to represent the upper and lower quartiles, and the *middle horizontal line* to represent the median. The *upper* and lower *whiskers* represent 5th and 95th percentile of the data. Variables acquired from the specimens included A, elastic modulus (*E*) from oscillatory nanoindentation, and levels of B, GAG; C, collagen; and D, elastin from biochemical assays. BAV-A was significantly more elastic in comparison with the control and DA. However, it was DA that had elevated collagen levels relative to the control. Only *E* was statistically different between the aneurysmal groups. Therefore, we can note that changes in micromechanical behavior between BAV-A and DA cannot be explained by only the levels of GAG and structural proteins within the tissue. Noticeably, BAV-A was overall more homogeneous than control and DA for majority of the in vitro measurements. *BAV-A*, Bicuspid aortic valve aneurysm; *DA*, degenerative aneurysm; *GAG*, glycosaminoglycan.

Overall, *E* (tissue stiffness) was greater in BAV-A than in DA and control groups, with greater homogeneity (interquartile range 22.64 for BAV-A 36.45 for DA and 43.65 for control) (Figure 3, *A*). *E* was greater for BAV-A than control (*P* = .022), and DA but not significant for the latter (*P* = .111). GAG and collagen levels were lower in BAV-A and control tissues relative to DA tissues but not significantly (Figure 3, *B* and *C*). Collagen levels were significantly greater in DA as compared with control tissue (*P* = .037) (Figure 3, *C*). Elastin levels were marginally greater in BAV-A relative to the control and DA but not significantly (Figure 3, *D*). A wider distribution of values was found in controls and DA compared with BAV-A for all measurements, except elastin levels. Elastin levels were highly variable in all 3 groups. We subsequently investigated the microstructural organization of elastin.

### Localized Loss of Elastin in BAV-A and DA Tissues

**Figure 4:**
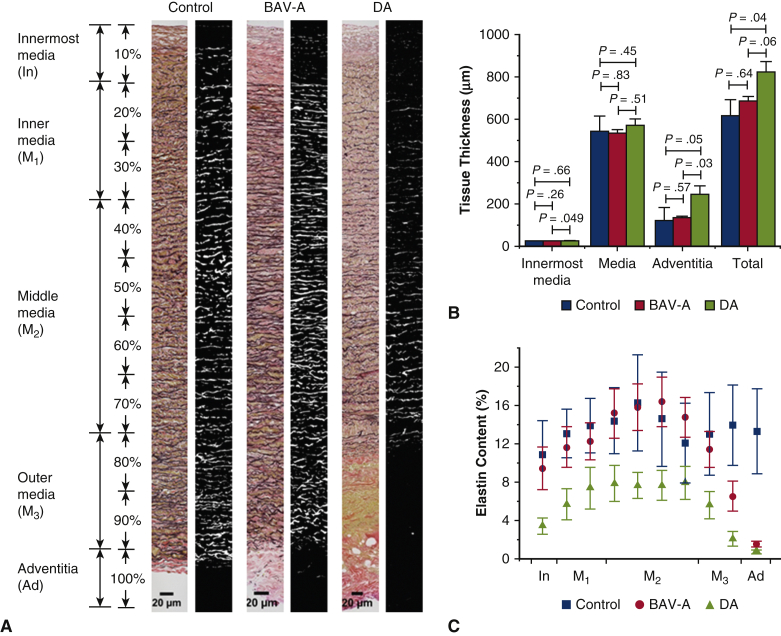
Quantitative analysis of tissue thickness and elastin content for control (n = 8), BAV-A (n = 15) and tricuspid aortic valve idiopathic DA (n = 15), expressed as mean ± standard error. A, Representative stitched original and mask image of the Verhoeff Van Gieson–stained aortic section for the 3 tissue groups (40× magnification). Tissue cross-sections were divided into 10 sections that covered innermost media (In), inner media (M_1_), middle media (M_2_), outer media (M_3_), and adventitia (Ad) layers. Over the tissue cross-section, B, tissue thickness, and C, elastin content of layers were quantified. Elastin content was quantified by the fraction area occupied by elastin relative to the entire area of the mask image. DA had a thicker innermost media and adventitia layer relative to BAV-A. More elastin was present in control and BAV-A compared with DA. Both BAV-A and DA had an extensive reduction in content between middle media and adventitia. Although there are thickness differences in the tissue layers between the aneurysmal groups, it was found that only these groups had a similar percentage reduction in elastin content toward the outer portion of the aortic wall. Scale bar: 20 μm. All *P* values for elastin content are presented in Table E2. *BAV-A*, Bicuspid aortic valve aneurysm; *DA*, degenerative aneurysm.

Elastin microstructural organization was measured for all cohorts (Figure 4, *A*). Before this, layer thickness, and elastin content (referring to the percentage of elastin per tissue section, see Figure 2) was measured. Medial thickness did not differ among groups (*P* > .34) (Figure 4, *B*). However, the thickness of the innermost media and adventitial layer was significantly lower in BAV-A compared with DA (*P* = .049, and *P* = .033, respectively). DA tissue was thicker overall compared with BAV-A and controls (*P* = .057, and *P* = .039, respectively). Elastin content was significantly greater for BAV-A than DA over the entire aortic cross-section, regardless of tissue thickness (*P* = .007-.047) (Figure 4, *C*). In fact, BAV-A had >2× elastin than DA in most tissue sections. Although BAV-A and DA contained different amounts of elastin, between M_2_ and adventitia, the content was reduced by 91% in both groups (from 16.4 ± 10.0% to 1.5 ± 1.2%, and from 7.6 ± 5.9% to 0.7 ± 0.8% respectively). Initially, BAV-A tissues followed a similar trend to the control tissues, whereby the elastin content increased between innermost media and M_2_. However, from M_3_ to adventitia, it was constant in the control tissue (from 12.0 ± 11.7% to 13.3 ± 12.6%) but reduced significantly for BAV-A tissue. In most of the regions, control tissues contained significantly more elastin than DA tissues (*P* = .0004-.041).

### Extensive Elastin Delamination in DA Relative to BAV-A

**Figure 5:**
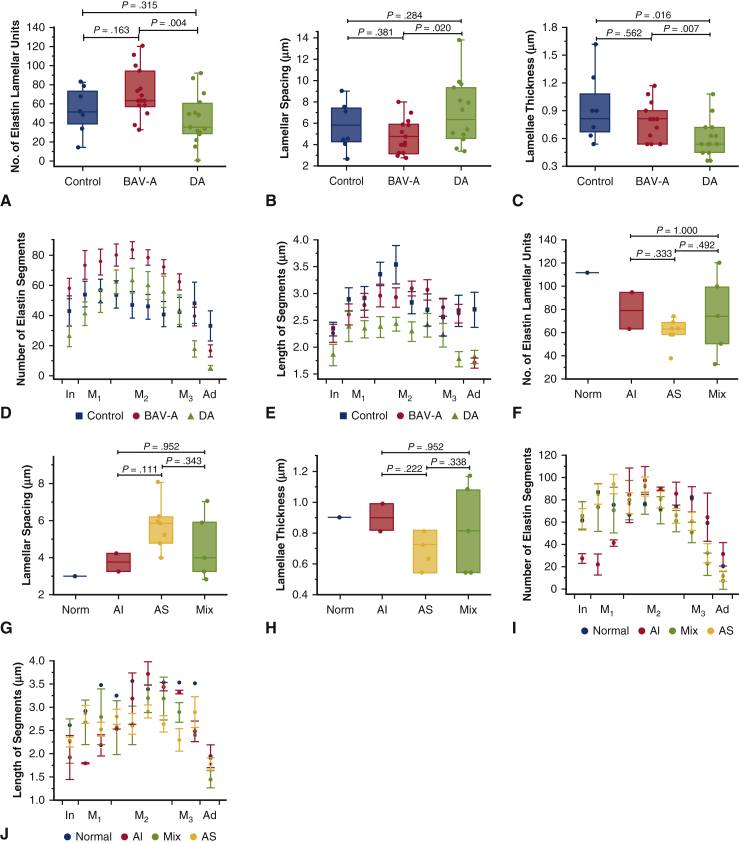
Quantitative analysis of elastin microstructure within control (n = 8), BAV-A (n = 15) and tricuspid aortic valve idiopathic DA (n = 15) aortic tissues. Quantitative analysis of elastin microstructure within bicuspid aortic valve aneurysm aortic tissues with normal valve function (Norm; n = 1), AI (n = 2), AS (n = 7), and a mixture of AI and AS (Mix; n = 5) are also shown F-J. Data in A-C and F-H were represented as *box plots* and data overlaid with *upper* and *lower borders* of the box to represent the upper and lower quartiles, and the *middle horizontal line* to represent the median. The *upper* and *lower whiskers* represent 5th and 95th percentile of the data. A, The number of elastin lamellar units; B, the lamellar spacing; and C, the thickness of the elastic lamellae were measured within the media. D, The number of elastin segments; and E, the length of the segments were measured over the entire tissue cross-section, expressed as mean ± standard error. Tissue cross-section was divided into 10 sections that covered innermost media (In), inner media (M_1_), middle media (M_2_), outer media (M_3_), and adventitia (Ad) layers. BAV-A had significantly more lamellar units with narrow lamellar spacing compared with DA. Control tissue had less lamellar units with wider lamellae spacing relative to BAV-A, although more units with narrower spacing relative to DA. DA had thinner lamellae compared with BAV-A and control. BAV-A and the control have similar elastin lamellae properties, although the number of segments differed between the groups. There were fewer short segments in DA relative to BAV-A and the control. Overall, we can note that although elastin segments were more uniformly distributed in the control, these segments were more compacted within the media of the aneurysmal tissues. All *P* values for elastin segments are presented in Tables E2 and E4. F-J, The same analysis as presented for A-E but within the BAV-A group only separating patients based on valve function. *BAV-A*, Bicuspid aortic valve aneurysm; *DA*, degenerative aneurysm; *AI*, aortic insufficiency; *AS*, aortic stenosis.

BAV-A tissues had the greatest number of lamellar units and DA tissues had the least (71.7 ± 25.4, and 44.1 ± 25.7, respectively) (Figure 5, *A*). There was no statistical difference in these parameters between the control and aneurysmal tissues (*P* > .16). However, on average control tissues had more lamellar units with narrower lamellae spacing compared with DA and less units and wider spacing compared with BAV-A. As there were more lamellar units for BAV-A, within a similar-sized medial space, the lamellar spacing was significantly reduced relative to DA tissues (*P* = .004) (Figure 5, *B*). Hence, BAV-A had a highly compacted elastin lamellae within the media. In agreement with the results presented on elastin content, both control and BAV-A tissues had significantly thicker elastic lamellae (0.84 ± 0.33 μm, and 0.76 ± 0.18 μm respectively) compared with DA tissues (0.60 ± 0.17 μm) (*P* < .028) (Figure 5, *C*). Multiple regression analysis showed that age was significantly associated with elastin lamellar thickness (Table E3).

We also measured the number of elastin segments for each group. Across the entire aortic tissue cross-section, the number of elastin segments was not uniformly distributed for both aneurysmal cohorts compared with controls (Figure 5, *D*). More elastin segments were found in M_2_ compared with the other regions for BAV-A and DA. From the innermost media to M_1_, there was a 31% increase of segments in both groups; control (from 43.0 ± 28.1 to 56.3 ± 21.5) and BAV-A (from 57.8 ± 25.7 to 75.5 ± 33.3, respectively), whereas in DA, 86% increase (from 26.5 ± 27.3 to 49.4 ± 28.9). At M_2_, it was noted that the number of segments were constant in each group. Subsequently, there was a larger reduction of segments for BAV-A by 79% (from 78.3 ± 20.2 to 16.6 ± 14.2) and DA by 91% (from 59.8 ± 35.3 to 5.6 ± 6.9), whereas the control had a small reduction of 28% (from 46.1 ± 23.0 to 33.4 ± 27.7). These findings suggest that elastin is more uniformly distributed in the controls across the aortic wall but much more localized in the aneurysm tissues, particularly within the central media. Interestingly, it was found that BAV-A had more elastin segments throughout the tissue compared with DA; in particular, it was significant between innermost media and M_1_ (*P* = .002-.009), and adventitia regions (*P* = .010-.011). Control tissues had significantly fewer segments compared with BAV-A tissues, within M_2_ and M_3_ regions (*P* < .043) and significantly more segments relative to DA tissues at the innermost media and adventitia regions (*P* < .045).

Lastly, we examined the length of the elastin segments. Overall, control tissues had longer elastin segments compared with BAV-A (*P* > .11) and DA (*P* = .003-.032) tissues (2.8 ± 0.4 μm, 2.7 ± 0.4 μm, and 2.2 ± 0.3 μm, respectively) (Figure 5, *E*). Between innermost media and M_2_, there was a 51% increase in the segment length in the control tissues (from 2.3 ± 0.4 μm to 3.5 ± 1.0 μm), whereas there was a 30% increase in BAV-A (from 2.3 ± 0.7 μm to 2.9 ± 0.7 μm) and DA tissues (from 1.8 ± 0.8 μm to 2.4 ± 0.5 μm). Between M_2_ and M_3_, the segment length decreased by 28% in the control tissues but was found to be constant in the aneurysmal tissues. Interestingly, an opposite trend was observed in the adventitia; segment length was constant in control tissues from M_3_ to the advential edge, whereas the aneurysmal tissues had a reduction in length of 38% for BAV-A (from 2.7 ± 0.6 μm to 1.7 ± 0.4 μm) and 18% for DA (from 2.2 ± 0.9 μm to 1.8 ± 0.5 μm). The segments were longer in BAV-A relative to DA across the tissue cross-section; in particular, significant differences were observed at innermost media and M_3_ region and the adventitia (*P* = .001-.035). In control and BAV-A tissues, across most of the tissue cross-section, segment lengths were similar. Control tissues had significantly longer segments compared with DA tissues (*P* = .003-.032).

### Microstructural Differences Among Patients With BAV-A With Different Valve Function

**Figure E1:**
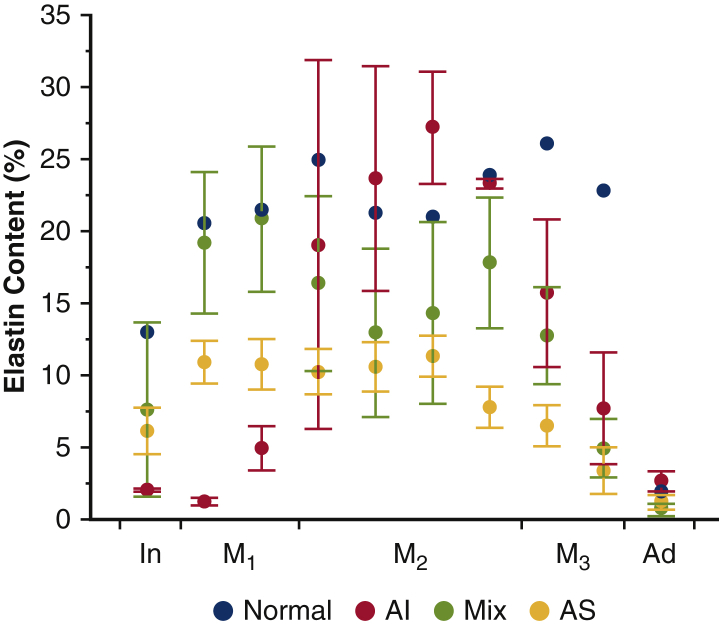
Microstructural differences of the aorta amongst BAV-A patients with different valve function. Elastin content within bicuspid aortic valve aneurysm aortic tissues with normal valve function (Norm; n = 1), AI (n = 2), AS (n = 7), and a mixture of AI and AS (Mix; n = 5), expressed as mean ± standard error of mean. *AI*, Aortic insufficiency; *AS*, aortic stenosis.

Patients with BAV-A were split according to the valve function, and the observed microstructural differences are shown in Figure 5, *F* to *J*. No significant differences were found between the types of valve function, which could be due to an uneven, small sample size for each group. However, patients with AI valves had more lamellar units, thicker lamellae, and narrow lamellar spacing compared with those with AS. Additional data are shown in Figure E1 and Table E4.

## Discussion

**Figure 6:**
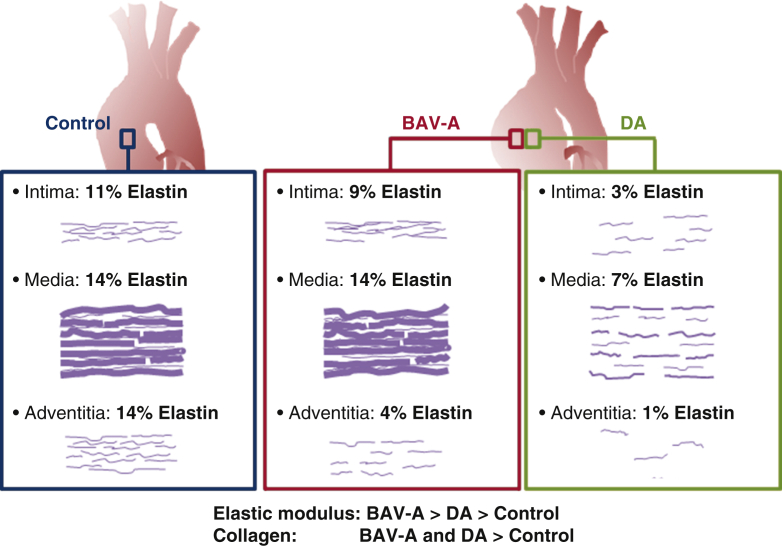
Summary schematic highlighting the phenotypic differences between the control, BAV-A, and tricuspid aortic valve idiopathic DA. Over the tissue cross-sections, there was more elastin content in the control and BAV-A tissues relative to DA tissues. Although control and BAV-A tissues appear to be similar microstructurally, subtle differences appear in the media and adventitia layers. BAV-A tissues had more elastin content, lamellar units, and elastin segments, thicker lamellae, and narrower lamellar spacing compared with DA tissues. These microstructural differences along with differences in collagen level could explain for the elevated micromechanical response. *BAV-A*, Bicuspid aortic valve aneurysm; *DA*, degenerative aneurysm.

In this study, we have used a novel micromechanical–microstructural approach to demonstrate the differential phenotype in patients with BAV-A compared with patients with DA. We found that dilation in both aneurysm groups is underlined by very different matrix degradation processes (Figure 6). We are the first to report mechanical properties, biochemical data, and histology for the same tissue sample. This was possible with nanoindentation, which allows localized, high-resolution measurements of tissue stiffness, nondestructively. Nanoindentation has been validated for aortic tissue characterization previously^16^ and allows correlation with localized microstructure. Recent work suggests highly localized alterations in aortic microstructure occur in dissection and ruptures^17^ affecting localized mechanical properties.^18^ This motivated us to examine these properties in parallel with biochemical measurements on the same samples, thereby allowing elastin microstructure-property relationships to be explored.

We found that *E* was significantly lower in the control group, although there were no significant differences in GAG, collagen, or elastin levels in BAV-A relative to controls (Appendix E1, Table E5). Stiffening of the aortic wall is associated with aneurysms.^19^ Of the aneurysm groups, BAV-A tissues were more homogenous than DA tissues. This was expected, given that the underlying cause for BAV-A is the aortic valve configuration and associated abnormal hemodynamics.^20^^,^^21^ In contrast, DA is associated with numerous factors and the initial triggering event is undetermined.^22^

Our aneurysmal cohorts were well-matched for key clinical parameters, including age and preoperative aortic size. Matrix protein levels for BAV-A and DA tissues could not explain differences in micromechanical behavior. This behavior could be addressed by understanding elastin distribution and microarchitecture. We hypothesized that elastin microstructure may play a role in distinguishing between the etiology of AsAA.

Previous studies have used semiquantitative analysis to grade elastin degradation within tissues.^23^^,^^24^ To the best of our knowledge, we are the first to present quantitative analysis of elastin distribution over the entire tissue cross-section in ascending aortic tissue. BAV-A tissues had more lamellar units, thicker elastin lamellae, and narrower lamellar spacing compared with DA tissues. These findings are consistent with the greater *E* in the BAV-A. A notable finding was the spatial distribution across the aorta wall of elastin for the different groups. Overall, the control and BAV-A tissues had more elastin content compared with DA tissues. BAV-A and the control tissues have similar elastin content around the central portion of the media. The striking difference in the aneurysm groups was in the inner (innermost medial region) and outer regions (adventitial region) of the wall, with a localized loss of elastin most pronounced in DA. Although severe elastin loss was not observed in BAV-A compared with DA, we noted a greater number of elastin segments within the tissue, possibly suggesting that elastin was increasingly fragmented in BAV-A.

Elastin degradation in BAV-A could be related to molecular pathology or altered hemodynamics.^5^ BAV-A affects elastin alignment within the aortic wall; hence, we propose that the altered hemodynamic pressure could compact medial elastin fibers. Increased regional wall shear stress has also been associated with greater medial elastin degradation in BAV-A.^25^ Therefore, highly compact, long, and aligned elastin segments within the medial layer of BAV-A tissue could explain the loss of aortic wall compliance. Biochemically, elastin degradation is related to matrix metalloproteinase (MMP) activity. Despite being highly resistant to proteolysis, elastin fibers can be degraded by certain MMPs,^26^ for example, MMP-2 levels were found to be significantly elevated in AsAAs relative to CABG controls.^27^ MMP-2 and MMP-9 expressions have been found to be significantly increased in DA relative to BAV-A.^28^^,^^29^ Conversely, MMP-2 expression was shown to be significantly elevated in patients with BAV relative to those with TAV.^30^ Fibrillin-1 content is also significantly reduced in BAV relative to TAV.^30^ Fibrillin-1 plays an important role in the elastic fiber system. Hence, we can postulate that elastin disruption within the adventitia and innermost media of the aortic wall along with its inability to regulate tissue development and elasticity may be the key in distinguishing the onset of ascending aneurysm formation.

### Study Limitations

**Figure E2:**
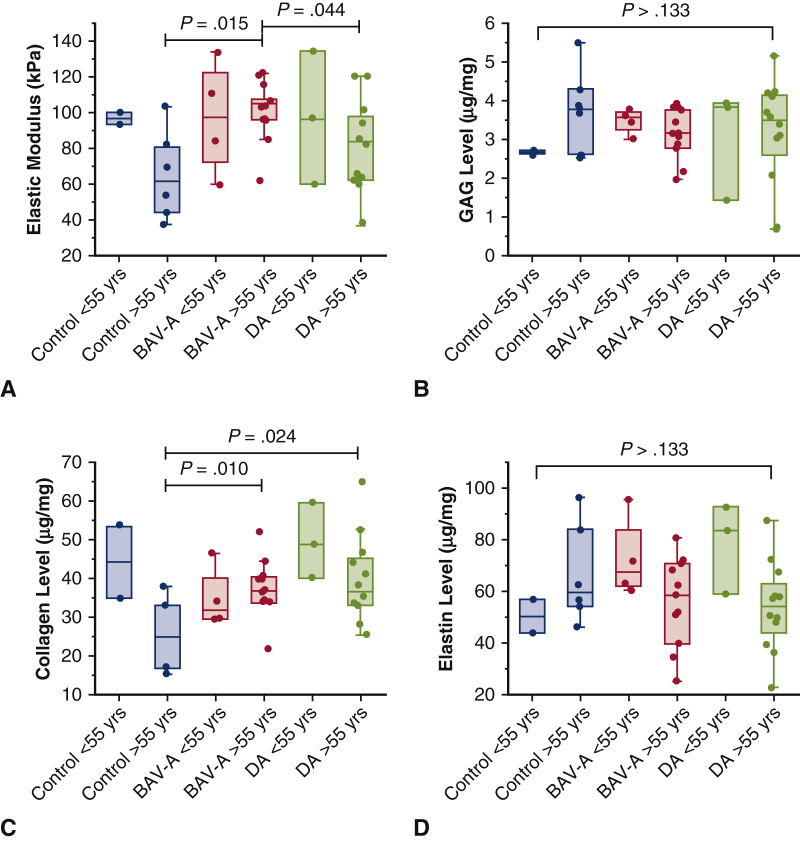
Micromechanical and biochemical data for control, BAV-A and DA, according to age <55 years old, and age >55 years old. Variables acquired included A, elastic modulus; B, GAG level; C, collagen level; and D, elastin level. *BAV-A*, Bicuspid aortic valve aneurysm; *DA*, degenerative aneurysm; *GAG*, glycosaminoglycan.

There were several limitations with this study. First, our aneurysmal patients were not indexed to body size before surgical intervention. “Aortic size index” may be useful to stratify patients instead of absolute aortic size for future studies. Second, we were limited to CABG punch biopsies as controls. In future, we would seek to use non-aneurysmal BAV/TAV tissue. Third, precise surgical sites (ie, greater/lesser curvature) could not be identified. Hence, our data may be a combination of both the greater and lesser curvature. Greater precision could be incorporated in tissue siting in the future. Fourth, collagen levels were significantly altered in DA, but detailed microstructural analysis of the collagen fiber organization is beyond the scope of this paper. Fifth, the nanoindentation method used assumes linear viscoelastic behavior; hence, the constitutive tissue behavior cannot be captured. Finally, some studies have reported differences in BAV-A aortopathy in young and old patients. We did not separate groups based on age to avoid low ‘n’ numbers. However, our trends remain the same but with greater significance if we remove the younger patients (see Figure E2).

## Conclusions

We found that there is a loss of tissue compliance in BAV-A and DA proximal aortic tissue relative to controls, with BAV-A being the stiffest. BAV-A and DA can be separated into 2 distinct groups. Loss of tissue compliance in the aneurysmal tissues was unrelated to elastin levels but rather the arrangement of elastin fibers (Video 1). The randomness in arrangement with short, thin elastin segments was most profound in DA, particularly in the innermost media and adventitia. BAV-A appeared to be much more intact structurally but with the elastin much more compacted than DA or controls. Although current treatment guidelines for operative intervention according to size do not differentiate between BAV-A and DA, further studies looking at the different subsets of BAV-A are necessary to differentiate whether certain groups of patients are at an increased risk of rupture at lower diameter thresholds. Our study suggests that BAV and DA aortopathy should not be grouped together, given the stark differences between them.

**Video 1:**
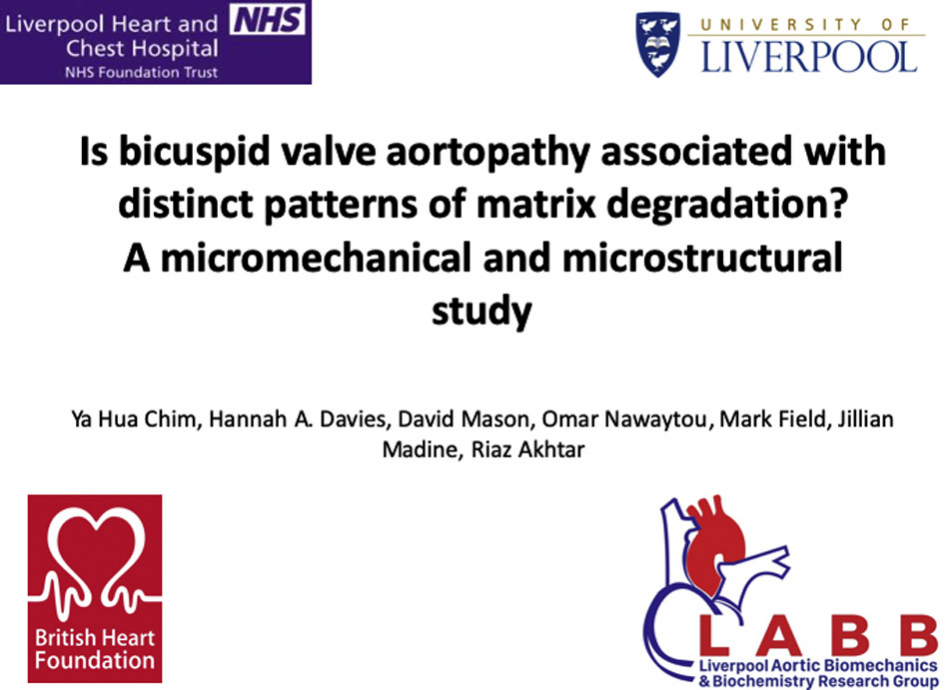
Elastin microstructural organisation has a unique pattern in bicuspid aortic valve aneurysm relative to idiopathic aortic aneurysm. Video available at: https://www.jtcvs.org/article/S0022-5223(19)31891-4/fulltext.

### Conflict of Interest Statement

Authors have nothing to disclose with regard to commercial support.

## Figures and Tables

**Table:** 

GAG	Freeze/thaw (n = 1)	Freeze/thaw (n = 2)	*P* value
BAV-A	3.0 ± 0.6	3.1 ± 0.6	.53
DA	3.5 ± 1.5	2.9 ± 1.3	.11

Data are displayed as mean ± standard deviation. *P* value for Mann–Whitney. *GAG*, Glycosaminoglycan; *BAV-A*, Bicuspid aortic valve aneurysm; *DA*, degenerative aneurysm.

**Table E1:** A list of control tissues that was used for each experiment, with clinical characteristics

Tissue identification	Age, y	Sex	Biomechanical	Biochemical	Elastin quantification
05-00068-16	63	M	√	√	√
05-00014-17	63	M	√	√	√
05-00035-17	61	M	√	√	√
05-00053-17	46	M	√	√	√
05-00057-17	49	M	√	√	√
05-00062-17	74	F	√	√	√
05-00063-17	64	F	√	√	X
05-00064-17	67	M	√	√	√
05-00087-17	51	M	X	X	√

*M*, Male; *F*, female.

**Table E2:** *P* values for the elastin properties between tissue groups, using an .05 significance level

Elastin quantification	Proportion of the tissue from innermost media to adventitia									
	10%	20%	30%	40%	50%	60%	70%	80%	90%	100%
Content										
BAV-A vs control	.872	.639	.708	.973	.757	.312	.298	.886	.183	.100
DA vs control	**.021**	**.024**	**.022**	**.041**	.053	.254	.435	.088	**.0004**	**.001**
DA vs BAV-A	**.010**	**.031**	**.021**	**.016**	**.007**	**.010**	**.029**	**.027**	**.008**	**.047**
No. segments										
BAV-A vs control	.247	.188	.225	**.034**	**.006**	**.011**	**.012**	.135	.766	.255
DA vs control	.166	.389	.468	.364	.204	.167	.185	.945	**.015**	**.001**
DA vs BAV-A	**.002**	**.009**	**.020**	.146	.073	.160	.153	.087	**.010**	**.011**
Length of segments										
BAV-A vs control	.527	.253	.402	.162	.111	.192	.239	.621	.986	**.006**
DA vs control	**.017**	**.023**	.054	**.007**	**.003**	.171	.691	.386	**.008**	**.032**
DA vs BAV-A	**.035**	.175	.191	.118	.100	**.001**	.059	.106	**.002**	.536

Significant values highlighted in bold. *BAV-A*, Bicuspid aortic valve aneurysm; *DA*, degenerative aneurysm.

**Table E3:** Multiple regression analysis on clinical characteristics with the in vitro measurements

Statistical model	Control		BAV-A		DA		Difference
	Regression coefficient	*P* value	Regression coefficient	*P* value	Regression coefficient	*P* value	
Elastic modulus							
Intercept	–729.714	.319	239.343	.083	122.650	.917	
Age	–10.102	.319	–0.683	.277	–0.388	.917	
BMI	–9.778	.319	–0.736	.277	–0.300	.917	
Preoperative aortic size	437.289	.319	–13.305	.277	–2.041	.917	
Sex	177.492	.319	–11.507	.277	–7.292	.917	
Cholesterol	18.450	.596	–50.297	.060	27.869	.917	
Hypertension	–31.292	.398	50.207	.060	–21.864	.917	
Family history of aneurysm	–	–	8.305	.58	4.399	.917	
GAG level							
Intercept	–11.780	.712	–3.446	.515	0.246	.969	
Age	–0.092	.712	0.017	.515	–0.012	.969	
BMI	–0.303	.712	0.022	.515	0.051	.969	
Preoperative aortic size	7.688	.712	0.583	.515	0.571	.969	
Sex	2.503	.712	–0.087	.783	0.387	.969	
Cholesterol	–4.404	.712	0.445	.579	–0.111	.969	
Hypertension	4.026	.712	0.226	.768	0.615	.969	
Family history of aneurysm	–	–	0.611	.515	–1.017	.969	
Collagen level							
Intercept	123.737	.200	36.708	.910	19.582	.864	
Age	–1.894	.200	0.019	.983	–0.118	.864	
BMI	–1.288	.200	0.155	.949	0.276	.864	
Preoperative aortic size	16.305	.451	1.249	.983	–2.837	.864	
Sex	19.387	.200	–10.402	.360	–1.688	.864	
Cholesterol	–25.515	.200	–0.161	.983	11.613	.864	
Hypertension	7.850	.200	–5.669	.910	–2.031	.864	
Family history of aneurysm	–	–	6.818	.910	14.320	.864	
Elastin level							
Intercept	–182.136	.952	77.001	.783	226.943	.240	
Age	0.060	.991	–0.572	.783	–0.994	.412	
BMI	–2.134	.952	0.337	.783	–2.380	.419	
Preoperative aortic size	67.217	.952	–8.961	.783	1.607	.847	
Sex	39.427	.952	1.379	.905	–7.028	.847	
Cholesterol	40.472	.952	–8.316	.783	–9.025	.847	
Hypertension	–20.104	.952	16.278	.783	–3.0147	.866	
Family history of aneurysm	–	–	22.495	.783	–9.858	.847	
Number of elastin lamellar units							
Intercept	135.765	.876	369.462	.403	227.951	.736	
Age	–1.321	.876	–1.391	.403	–0.252	.823	
BMI	–3.402	.876	–0.927	.680	–3.163	.736	
Preoperative aortic size	5.572	.979	–20.178	.650	–5.210	.823	
Sex	40.944	.876	–27.120	.403	–15.286	.823	
Cholesterol	–1.242 E17	.876	–2.229	.942	–7.506	.823	
Hypertension	1.242 E17	.876	–5.990	.942	–7.018	.823	
Family history of aneurysm	–	–	–16.026	.767	–6.356	.823	
Elastic lamellae spacing							
Intercept	–14.142	.866	–9.487	.670	–35.156	.196	
Age	0.064	.866	0.097	.670	0.133	.312	
BMI	0.425	.866	0.046	.670	0.666	.196	
Preoperative aortic size	2.515	.866	0.661	.670	–0.123	.855	
Sex	–3.839	.866	1.567	.670	1.971	.377	
Cholesterol	–4.849 E15	.866	1.198	.670	4.153	.377	
Hypertension	4.8489 E15	.866	–1.271	.670	0.913	.810	
Family history of aneurysm	–	–	0.713	.680	3.501	.377	
Elastin lamellar thickness							
Intercept	4.950	.922	2.397	.628	2.537	.016	
Age	–4.247 E4	.992	–0.006	.726	–0.014	.024	
BMI	–0.053	.922	-0.004	.726	–0.024	.109	
Preoperative aortic size	–0.716	.922	–0.096	.726	0.005	.883	
Sex	0.320	.922	–0.222	.628	0.023	.883	
Cholesterol	1.175 E15	.922	–0.111	.726	–0.034	.883	
Hypertension	–1.175 E15	.922	0.086	.726	–0.125	.582	
Family history of aneurysm	–	–	–0.129	.726	–0.110	.582	
Elastin lamellar thickness							
Intercept					1.682	.078	–33.7%
BMI					–0.017	.310	–29.2%
Preoperative aortic size					–0.068	.135	–1460%
Sex					–0.025	.840	–208.7%
Cholesterol					–0.093	.715	173.5%
Hypertension					0.062	.726	–149.6%
Family history of aneurysm					–0.082	.679	–25.5%

Multiple regression analysis revealed that for majority of the in vitro measurements, there was no significant association with the clinical characteristics. However, age was found to be significantly associated with elastin lamellar thickness. Further analysis on the difference between regression coefficients using with and without age multiple regressions revealed that age was indeed a confounder within this model. *P* values adjusted with the Benjamini–Hochberg method. *BAV-A*, Bicuspid aortic valve aneurysm; *DA*, degenerative aneurysm; *BMI*, body mass index; *GAG*, glycosaminoglycan.

**Table E4:** Elastin microstructural properties of aortic tissue from BAV-A with different valve function, control, and DA patients

Elastin quantification	BAV-A: Normal (n = 1)	BAV-A: AI (n = 2)	BAV-A: AS (n = 7)	BAV-A: Mix (n = 5)	Control (n = 8)	DA (n = 15)	*P* value
Number of elastin lamellar units	111.5	79 ± 15	63 ± 7	74 ± 49	51 ± 29	36 ± 26	.192
Lamellar spacing, μm	3.0	3.7 ± 0.5	5.9 ± 1.1	4.0 ± 2.7	5.9 ± 3.0	7.3 ± 4.8	.117
Lamellae Thickness, μm	0.90	0.90 ± 0.09	0.72 ± 0.23	0.81 ± 0.54	0.81 ± 0.29	0.54 ± 0.23	.602
Elastin content (%)							
10% portion	13.0	2.1 ± 0.1	6.2 ± 6.7	7.7 ± 10.0	8.0 ± 9.2	1.6 ± 6.3	.041
20% portion	20.1	1.3 ± 0.3	11.0 ± 5.2	19.2 ± 18.0	13.2 ± 10.9	4.0 ± 7.2	.031
30% portion	21.5	5.0 ± 1.5	10.8 ± 4.9	20.9 ± 16.9	12.8 ± 9.1	4.6 ± 6.0	.048
40% portion	25.0	19.1 ± 12.8	10.3 ± 3.2	16.4 ± 19.8	10.6 ± 8.9	6.0 ± 5.9	.152
50% portion	21.3	23.7 ± 7.7	10.6 ± 2.8	13.0 ± 16.1	11.8 ± 6.4	6.6 ± 6.8	.056
60% portion	21.0	27.2 ± 3.9	11.4 ± 1.8	14.4 ± 17.0	8.8 ± 11.0	7.3 ± 8.8	.089
70% portion	23.9	23.3 ± 0.3	7.9 ± 4.0	17.9 ± 16.0	9.2 ± 9.2	9.5 ± 10.8	.092
80% portion	26.1	15.8 ± 5.1	6.6 ± 4.7	12.8 ± 5.0	8.1 ± 12.0	2.8 ± 10.8	.110
90% portion	22.9	7.8 ± 3.9	3.5 ± 2.1	5.0 ± 3.1	10.8 ± 10.5	1.1 ± 2.9	.005
100% portion	2.0	2.7 ± 0.7	1.3 ± 1.3	0.7 ± 0.3	13.0 ± 12.8	0.4 ± 0.8	.027
Number of elastin segments							
10% portion	61.0	27.3 ± 4.3	63.0 ± 23.3	65.5 ± 12.0	41.5 ± 29.6	19.5 ± 30.5	.035
20% portion	87.0	21.8 ± 9.3	85.0 ± 19.5	73.0 ± 11.5	53.5 ± 24.4	37.5 ± 44.0	.019
30% portion	75.0	41.0 ± 3.0	94.0 ± 24.5	70.5 ± 8.5	55.5 ± 25.3	47.5 ± 33.3	.041
40% portion	66.0	84.0 ± 24.5	76.5 ± 42.3	79.5 ± 20.0	49.5 ± 38.9	70.0 ± 25.0	.348
50% portion	75.5	97.3 ± 12.3	92.0 ± 37.3	76.5 ± 24.0	46.3 ± 34.0	67.0 ± 36.0	.083
60% portion	72.0	89.5 ± 1.5	80.0 ± 23.8	70.5 ± 20.0	46.3 ± 29.3	73.5 ± 58.3	.149
70% portion	73.5	85.3 ± 10.3	65.5 ± 28.8	60.5 ± 4.5	33.8 ± 18.5	54.5 ± 72.3	.213
80% portion	82.0	80.3 ± 11.3	60.5 ± 34.5	50.5 ± 30.0	34.8 ± 30.0	41.0 ± 64.0	.382
90% portion	59.0	64.0 ± 21.5	32.0 ± 12.0	22.0 ± 26.0	43.8 ± 25.4	9.0 ± 24.8	.048
100% portion	13.0	31.0 ± 10.5	11.5 ± 20.0	7.5 ± 4.5	30.5 ± 28.5	3.0 ± 8.0	.014
Length of elastin segments, μm							
10% portion	2.61	1.91 ± 0.47	2.25 ± 0.31	2.26 ± 0.22	2.28 ± 0.37	1.73 ± 0.47	.115
20% portion	2.91	1.79 ± 0.02	2.85 ± 0.80	2.67 ± 1.63	3.02 ± 0.67	2.31 ± 0.83	.106
30% portion	3.48	2.17 ± 0.22	2.53 ± 0.55	2.79 ± 1.88	2.82 ± 0.58	2.28 ± 0.82	.225
40% portion	3.25	2.53 ± 0.00	2.80 ± 0.28	2.56 ± 2.18	3.46 ± 0.78	2.60 ± 0.94	.112
50% portion	3.57	3.19 ± 0.57	2.65 ± 0.38	2.61 ± 1.66	3.33 ± 0.64	2.31 ± 0.69	.054
60% portion	3.39	3.72 ± 0.25	2.91 ± 0.33	3.19 ± 0.62	2.75 ± 0.23	2.23 ± 0.97	.025
70% portion	3.55	3.45 ± 0.09	2.63 ± 0.51	3.19 ± 0.97	2.59 ± 1.10	2.56 ± 1.24	.201
80% portion	3.53	3.33 ± 0.05	2.29 ± 0.95	2.89 ± 0.37	2.53 ± 1.22	2.37 ± 1.20	.236
90% portion	3.52	2.48 ± 0.22	2.89 ± 1.24	2.41 ± 0.52	2.57 ± 1.10	1.81 ± 0.57	.022
100% portion	1.89	1.94 ± 0.25	1.77 ± 0.38	1.44 ± 0.56	2.70 ± 1.20	1.81 ± 0.76	.088

Data are displayed as median ± interquartile range; n represents the number of patients. *P*-value for Kruskal–Wallis test. *BAV-A*, Bicuspid aortic valve aneurysm; *AI*, aortic insufficiency; *AS*, aortic stenosis. *DA*, degenerative aneurysm.

**Table E5:** Quantitative data acquired for all patients, grouped by clinical condition

Measured parameter	Control (n = 8)	BAV-A (n = 15)	DA (n = 15)	*P* value
Micromechanical properties				
*E*, kPa	76.0 ± 43.7	103.4 ± 22.6	85.6 ± 36.5	.057
Biochemical levels				
GAG, μg/mg	3.2 ± 1.4	3.2 ± 0.7	3.6 ± 1.5	.844
Collagen, μg/mg	33.0 ± 18.5	36.6 ± 6.3	40.2 ± 14.4	.115
Elastin, μg/mg	57.4 ± 15.7	63.1 ± 19.8	58.6 ± 21.2	.855
Collagen: elastin	0.55 ± 0.37	0.59 ± 0.27	0.64 ± 0.41	.297
Tissue thickness				
Intima, μm	20.8 ± 2.8	17.4 ± 3.9	23.6 ± 8.4	.135
Media, μm	506.0 ± 192.9	509.5 ± 82.5	552.1 ± 168.4	.698
Adventitia, μm	75.0 ± 115.9	134.2 ± 59.1	184.3 ± 133.8	.043
Total, μm	629.3 ± 332.5	678.3 ± 131.0	794.4 ± 229.0	.061
Microstructural properties of elastin				
Number of elastin lamellar units	52 ± 29	64 ± 26	36 ± 26	.017
Elastic lamellae spacing, μm	5.9 ± 3.0	4.8 ± 2.3	7.3 ± 4.8	.103
Elastin lamellar thickness, μm	0.81 ± 0.29	0.81 ± 0.27	0.54 ± 0.23	.019

Data are displayed as median ± interquartile range; n represents the number of patients. *P* value for Kruskal–Wallis test. *BAV-A*, Bicuspid aortic valve aneurysm; *DA*, degenerative aneurysm; *E*, elastic modulus; *GAG*, glycosaminoglycan.

## Appendix E1 Materials

The composition of chemicals used for the biochemical assays have been listed as follows: papain solution (0.1 M sodium acetate, 2.4 mM ethylenediaminetetraacetic acid, 5 mM L-cysteine, pH 5.8), dimethyl methylene blue dye (38 μM 1-9 dimethyl methylene blue, 29 mM sodium formate, 43 mM formic acid, pH 3.5), diluent (2:1 propan-2-ol: deionized water), oxidant (0.05 M chloroamine T, 0.3 M sodium acetate, 0.1 M trisodium citrate dihydrate, 24 mM citric acid, 5 M propan-2-ol), and color reagent for collagen levels (1 M 1,3-dimethylbutylamine, 1.5 M perchloric acid, 70%, 14 M propan-2-ol).

### Biochemical Assays

For papain digestion, 50 mg of tissue was incubated in 500 μL of papain solution at 60°C until tissue was completely digested. For oxalic acid digestion, 7.5 mg of tissue was incubated in 750 μL of 0.25 M oxalic acid at 100°C for 1 hour, and subsequently the digested supernatant was collected. Hot oxalic acid treatment of residues was repeated another 4 times, using fresh oxalic acid each time. The extracts (200 μL) from each digestion were pooled together and used for the assay.

To determine sulfate glycosaminoglycan levels within the tissue, the papain digested samples were diluted with water (1:10) to make a total volume of 40 μL, which was added to 250 μL of dimethyl methylene blue dye, and subsequently absorbance reads were measured at 570 nm. Chondroitin sulfate C was used as a standard.

Collagen levels were determined by the concentration of hydroxyproline within the tissue. The samples were hydrolyzed, and 100 μL of papain digested samples were added to 100 μL of 12 M hydrochloric acid and autoclaved. These samples were subsequently lyophilized and resuspended with 2 mL of deionized water. A proportion of the sample was diluted with water (1:4) to make a total volume of 250 μL, which was added to 250 μL of diluent, and 250 μL of oxidant, and was kept at room temperature for 20 minutes. Freshly prepared color reagent was added and kept at room temperature for 14 minutes, followed by incubation at 70°C for 20 minutes. The samples were cooled before absorbance reads were taken at 550 nm. L-hydroxyproline was used as the standard. As hydroxyproline represents 14% of mature collagen, the readings were adjusted accordingly.

To determine α-elastin levels, 200 μL of pooled oxalic acid digested tissue was bound to elastin-dye complex, and subsequently dye was released from the elastin with dye dissociation reagent, according to Fastin Elastin Kit (Biocolor, Carrickfergus, UK). The recovered dye was collected before absorbance reads were measured at 513 nm and compared against the standard within the kit.

### Image Processing

A shallow-learning pixel classifier was trained using 6 images to create 2 labels (fiber and background). The batch processing function was used to classify the rest of the images and output segmentation masks. From the images, the thickness of layers and elastin content of each section within the tissue were measured. Elastin content was quantified by the fraction area occupied by elastin relative to the entire area. Second, to quantify the elastic lamellae within the medial layer, a single central profile plot was taken from each stitched mask image. From the plots, the thickness of the lamellae was acquired from the distance within a peak, while the spacing was measured from the distance between peaks. The number of lamellar units was calculated by subtracting the number of spaces measured by one, as this consists of 2 elastic lamellae sandwiched between inter-lamellar spacing. Finally, the elastin segments were measured automatically with an in-house macro (https://bitbucket.org/snippets/davemason/Ke9y69). For each portioned section, the number of elastin segments and their lengths were measured.

### Impact of Sample Preparation and Storage

All of our tissue was snap-frozen using liquid nitrogen and an isopentane slurry and was immediately transferred to a –80°C freezer. There is evidence in the literature to suggest that our approach does not affect the tissue biomechanical properties. For example, work on porcine thoracic aorta by Hemmasizadeh and colleagues^E1^ reported (2012) “Generally, in freezer storage, E increased and G∞ showed no significant change. In prolonged preservation (>1 week), the results of –20°C showed significant increase in E (20% after 3 weeks), while this increase for –80°C was not significant, making it a better choice for tissue cold storage applications.” In another study by Chow and Zhang^E2^ on bovine thoracic aortas also reported findings in agreement with Hemmasizadeh and colleagues but actually proposed that even lower temperatures maintain the tissue integrity and biomechanical properties: “Overall, freezing protocols (either –20°C or –80°C) are suggested over refrigeration (4°C) for maintaining the initial stress-strain behavior.” As the structural and mechanical properties of –80°C stored tissue does not change, it is suggestive that under the same conditions biochemical levels within the tissue could also be unaffected. Using the biochemical tests, we studied the freeze/thaw cycle of the aortic tissue collected directly from surgery (freeze/thaw, n = 1), and tissue collected after micromechanical testing (freeze/thaw, n = 2) on 10 patients. As shown within the data in the table seen below, our storage condition did not affect the tissue.

## Supplementary Data

Video 1 Elastin microstructural organisation has a unique pattern in bicuspid aortic valve aneurysm relative to idiopathic aortic aneurysm. Video available at: https://www.jtcvs.org/article/S0022-5223(19)31891-4/fulltext.
